# Development of an immunoaffinity chromatography and LC-MS/MS method for the determination of 6 zearalenones in animal feed

**DOI:** 10.1371/journal.pone.0193584

**Published:** 2018-03-05

**Authors:** Mi Jin Lee, Ho Jin Kim

**Affiliations:** National Agricultural Products Quality Management Service, Kimchun, Republic of Korea; Babasaheb Bhimrao Ambedkar University, INDIA

## Abstract

A novel and simple method for detecting 6 zearalenones in animal feed using liquid chromatography coupled with triple quadrupole mass spectrometry (LC-MS/MS) and immunoaffinity columns (IAC) was developed. The chromatographic peaks of the 6 zearalenones were successfully identified by comparing their retention times and mass spectrum with reference standards. The mobile phase was composed of mobile phase A (water) and B (0.5% formic acid in ACN). Method validation was performed with linearity, sensitivity, selectivity, accuracy and precision. The limits of detection (LODs) for the instrument used to study zearalenones ranged from 0.3 to 1.1 μg/kg, and the limits of quantification (LOQs) ranged from 1.0 to 2.2 μg/kg. Average recoveries of the 6 zearalenones ranged from 82.5% to 106.4%. Method replication resulted in intra-day and inter-day peak area variation of <3.8%. The developed method was specific and reliable and is suited for the routine analysis of zearalenones in animal feed.

## Introduction

Zearalenone (ZON) is fungal toxin, a harmful substance for feed. The worldwide spotlight is shed upon its contamination of human food and animal feed. It is known to have critical influence not only on humans but also on livestock. ZON is fungal toxin that is produced by *Fusarium graminearum*, *Fusarium culmorum*, and other pathogens in corn, corn-derivatives, feeds, grains, and other food[1, 2]. ZON has α-zearalanol(α-ZAL), β-zearalanol(β-ZAL), α-zearalenol(α-ZOL) and β-zearalenol(β-ZOL), zearalanone(ZAN), which are isomer and metabolite, and these six types are called Zearalenone group (ZEN)[3].

**Fig 1:**
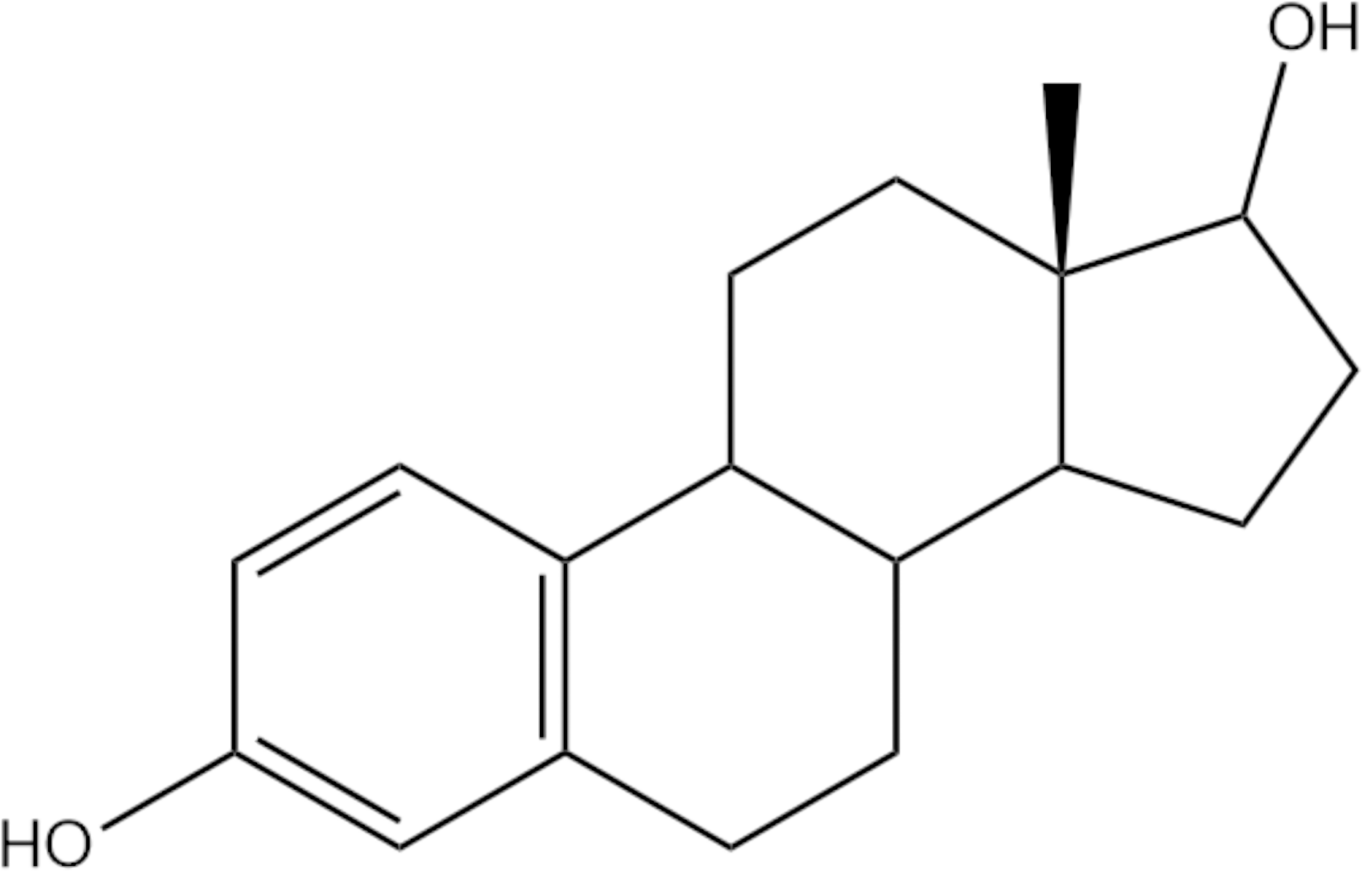
Structure of 17-estradiol, a similar chemical substance to ZEN.

ZON is metabolized mainly to a-ZOL and b-ZOL. Both metabolites are metabolized to a-ZAL and b-ZAL, respectively. ZAN is also metabolized y ZON, a-ZAL and b-ZAL, and the six isomers on matabolic pathway are correlated with each other. The chemical structures of ZON and ZON metabolite are similar to 17-estradiol (E2) and other natural estrogens (Fig 1). Therefore, competitive bonding to estrogen acceptors is conducted due to the action of environmental hormones. Relative estrogenicity of ZON and its metabolites was determined in comparison with E2 (92% for α-ZOL, 18% for α-ZAL, 3.5% for β-ZAL, 1% for ZON and 0.44% for β-ZOL). Regardless of its low acute toxicity, ZEN is considered hepatotoxic, hematotoxic, immunotoxic, genotoxic, teratogenic and carcinogenic in many mammals[4].

Currently, there exists an analysis method for ZON in feeds, but there are no analytical studies on its isomers. ZON exists in trace quantities at the ppb level in feeds, thus there is a possibility that analytical error may lead to a change in outcome. For more accurate and precise analysis, it is necessary to develop an analysis method for the 6 isomers.

Various methods have been reported for the characterization of ZEN, such as high performance liquid chromatography (HPLC)[2, 5], liquid chromatography (LC) coupled with mass spectrometry (MS)[6], LC coupled with MS/MS[7–10], and gas chromatography(GC) coupled with MS[11–14]. LC-MS and LC-MS/MS, which provide information about the molecular mass and structural features of components, are considered more useful than other methods for the separation, identification, and quantification of the characteristic ZEN compounds.

In this study, ZEN in animal feed (α-ZAL, β-ZAL, α-ZOL, β-ZOL, ZAN and ZON) were analyzed simultaneously. Simultaneous identification was initially developed using the IAC and LC-MS/MS for the six types of ZEN, α-ZAL, β-ZAL, α-ZOL, β-ZOL, ZAN and ZON. A novel analytic method is therefore reported, which is suitable for detecting and quantifying the ZEN present in animal feed, while also aiding in the management of feed standards.

## Experimental

### Samples

The ZEN present in animal feed were targeted in this study. Animal feed was used after directly acquiring it from a local farm and old feed was used rather than feeds currently sold in the market. These samples were obtained from farms in five different cities. A total of 75 animal feed samples were used,. All dried samples were pulverized into fine powders (HMF-100; HANIL Electric Co., Seoul, Korea). The pulverizer was set at a maximum speed of 22,000 rpm to give fine powders ranging in size from 400 to 1000 μm. All samples were stored at 4°C.

### Chemicals and reagents

HPLC grade acetonitrile and methanol were purchased from Merck (Darmstadt, Germany). Phosphate buffered saline(PBS) was purchased from Sigma (St. Louis, Mo, USA). ZEN(α-ZAL, β-ZAL, α-ZOL, β-ZOL, ZAN, ZON) were purchased from Sigma (St. Louis, Mo, USA). As purification IAC, immunoaffinity columns for Zearalenones (IAC-ZER) was purchased from Clover (Irvine, USA). All other organic chemicals and organic solvents were reagent grade or higher.

### Standard preparation

The ZEN was dissolved with acetonitrile in a 10 mL volumetric flask to produce stock standard solutions. The concentrations of stock solutions ranged from 1.0 to 10 mg/mL. The appropriate volumes of each stock solution were mixed together, and then diluted serially to prepare the working standard solutions. All solutions were stored under refrigeration.

### Sample preparation

The samples were prepared for extraction by IAC. Five grams of homogenized sample were place into a centrifuge tube and then mixed with 50 mL of solution (ACN:D.W, (80/20, [v/v])). The mixture was homogenized for 30 min, and sonicated (S450H; HUCOM system, Suwon, Korea) for 30 min at room temperature. It was then centrifuged at 3000 rpm for 5 min. The supernatant (10 mL) was mixed with 20 mL of PBS and filtered (Whatman, No. 4). 10 mL of the filtrate was passed through the IAC-ZER. 10 mL of water was passed through the IAC-ZER. The sample was eluted from the IAC-ZER by passing 2 mL of methanol, and all eluted samples were collected in a new test tube. The sample eluate was evaporated under a gentle stream of nitrogen at 50°C and the residue was dissolved in 500 μL of solution (40% ACN) and filtered through a 0.22 μm disposable filter prior to chemical analysis. All measurements were performed in triplicate.

### HPLC-MS/MS analysis

HPLC analysis was performed on an Agilent 1200 HPLC system coupled with an Agilent 6460 triple quadrupole mass spectrometer (Agilent Technologies, Diegem, Belgium) in positive ion mode ([M+H]^+^) that was equipped with an Agilent 1260 series HPLC system. The chromatographic column used was a UK-C18 column (150 x 3 mm, 3 μm) (Tokyo, Japan) at 40°C. The mobile phase was filtered through a 0.45 μm membrane filter (Millipore, Milford, MA, USA) and degassed under vacuum. The mobile phase was composed of mobile phase A (water) and B (0.5% formic acid in ACN) with the following gradient elution: 0 min, 41% B; 0–5 min, 41% B; 5–35 min, 42.2% B; 35–40 min, 42.2% B; 40–40.1 min, 41% B; 40.1–45 min, 41% B. The sample injection volume was 10 μL, and the flow rate was set at 0.5 mL/min. The optimized electrospray ionization conditions were as follows: gas temperature, 150°C; gas flow, 9 L/min; sheath gas temperature, 350°C; sheath gas flow, 11 L/min; capillary voltage, 3000 V. Ionization condition and multiple reaction monitoring(MRM) for quantitative analysis are as illustrated in the Table 1

**Table 1:** Multiple reaction monitoring (MRM) conditions for the six Zearalenone group (ZEN) compounds by LC-MS/MS.

Compound Name	Q1 (m/z)	Q3 (m/z)	Fragmentor	Collision Energy	Cell Accelerator Voltage	Polarity
α-zearalanol (α-ZAL)	323.2	287.4	50	10	7	positive
		305.2	50	0	7	positive
β-zearalanol (β-ZAL)	323.2	305.1	60	0	7	positive
		287.1	60	10	7	positive
α-zearalenol (α-ZOL)	321.9	303.1	50	5	7	positive
		285.2	50	5	7	positive
β-zearalenol (β-ZOL)	321.2	303.2	60	0	7	positive
		285.1	60	5	7	positive
Zearalanone (ZAN)	321.2	303.2	60	5	7	positive
		285.1	60	10	7	positive
Zearalenone (ZON)	319.2	301.1	60	0	7	positive
		283.1	60	5	7	positive

### Method validation

Method validation was performed according to the guidelines set by the International Conference on Harmonization (ICH, 2005)[15] and the International Union of Pure and Applied Chemistry (IUPAC, 2002)[16]. The method was validated for linearity, sensitivity, selectivity, accuracy and precision.

## Results and discussion

### Method validation

The LC-MS/MS method had been developed by using IAC was validated to verify that its performance was compatible with the required performance for routine ZEN analysis in animal feeds. A few performance characteristics were measured, including selectivity, linearity, sensitivity, accuracy, and precision[17, 18].

**Fig 2:**
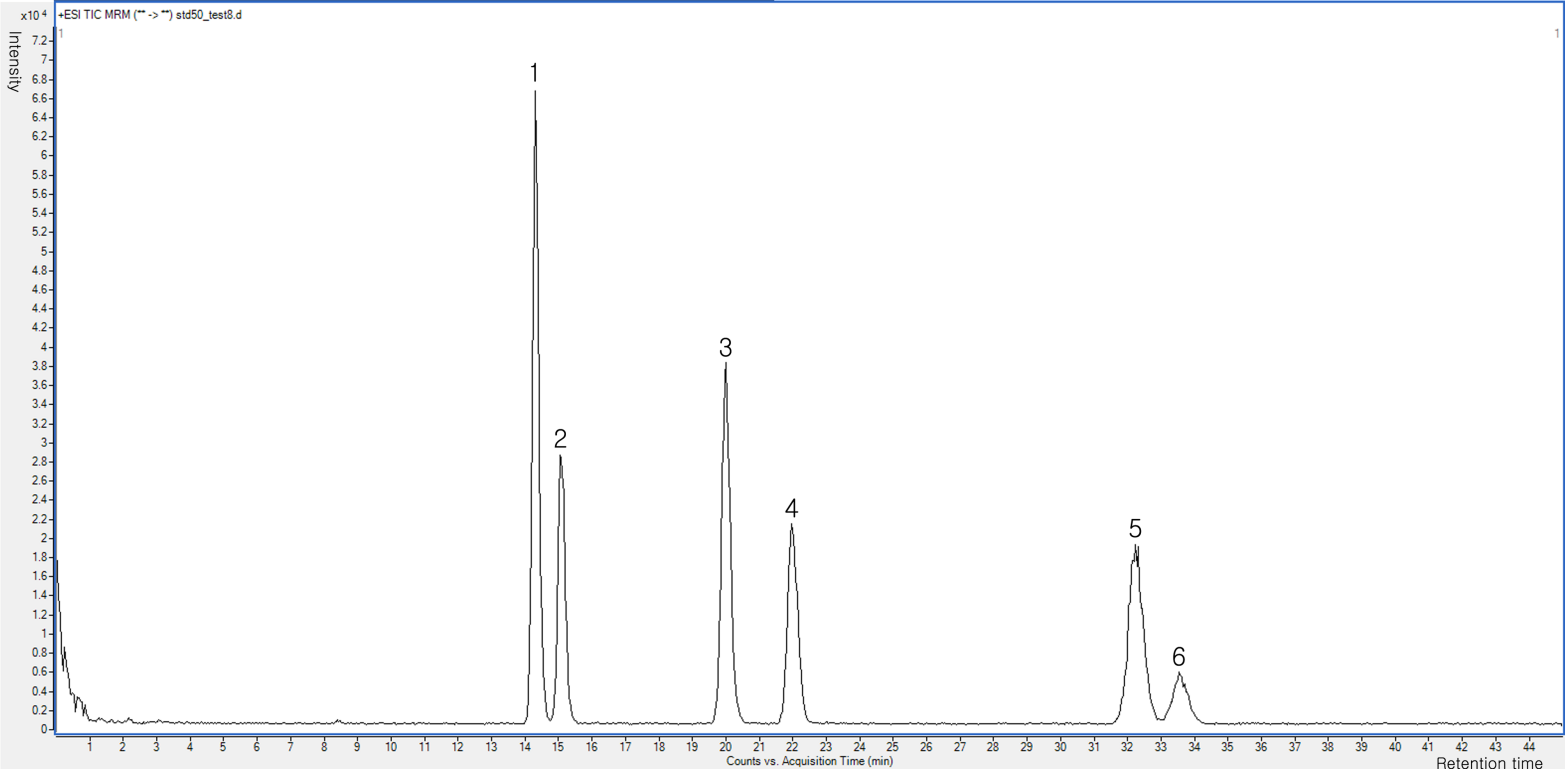
LC-MS/MS chromatograms of in a standard mixture of 6 zearalenone metabolites. Standard mixture of the six types of zearalenone group was put to 2 μL injection. (1) β-zearalanol(β-ZAL), (2) β-zearalenol(β-ZOL), (3) α-zearalanol(α-ZAL), (4) α-zearalenol(α-ZOL) (5) zearalanone(ZAN), (6) zearalenone(ZON).

The selectivity was determined without interfering on the chromatographic window. The chromatograms of the ZEN indicate the separation of all six compounds in <35 min successful in high good resolution (1.32–18.22) and asymmetry (0.92–1.50) as shown in Fig 2 and Tables 2–4, thereby, it displays the satisfactory selectivity of this LC-MS/MS system.

**Table 2:** Validation parameters of the developed LC-MS/MS method.

Compound	RT^a^	slope	intercept	r^2^^b^	LOD^c^ (μg/kg)	LOQ^d^ (μg/kg)	Rs^e^	Symm^f^
α-zearalanol (α-ZAL)	20.01	26070	-20082	0.9994	0.4	1.3	-	0.92
β-zearalanol (β-ZAL)	14.30	36473	-1164139	0.9999	0.3	1.0	1.55	0.97
α-zearalenol (α-ZOL)	21.96	50152	-1724819	0.9998	0.6	2.2	8.77	0.99
β-zearalenol (β-ZOL)	15.03	11336	-416581	0.9999	0.6	2.1	2.90	0.93
Zearalanone (ZAN)	32.23	12803	-482454	0.9997	0.6	2.0	18.22	1.21
Zearalenone (ZON)	33.54	16172	-440945	0.9998	1.1	3.1	1.32	1.50

^a^ Retention time (min).

^b^ Coefficients of correlation

^c^ Limit of detection, the lowest analyte concentration that produces a response detectable above the noise level of the system.

^d^ Limit of quantification, the lowest level of analyte that can be accurately and precisely measured.

^e^ Resolution.

^f^ Symmetry.

**Table 3:** Intra- and inter- day precision and accuracy data of Zearalenone group(ZEN) determination (n: Independent replicates).

	Nominal (conc, μg/kg)	Intra-day (n = 6)		Inter-day (n = 9)	
		Mean measured concentration (μg/kg)	RSD (%)	Mean measured concentration (μg/kg)	RSD (%)
α-ZAL	2	1.92	2.5	1.84	3.2
	20	19.40		19.16	
β-ZAL	2	1.88	3.8	1.90	2.7
	20	19.00		19.14	
α-ZOL	2	1.91	2.2	1.90	1.8
	20	19.35		19.44	
β-ZOL	2	1.88	3.2	1.90	2.4
	20	19.31		19.33	
ZAN	2	1.91	1.8	1.91	2.3
	20	19.51		19.20	
ZON	2	1.93	1.4	1.93	1.8
	20	19.66		19.52	

**Table 4:** Recovery of Zearalenone group(ZEN) in animal feeds.

Compound	Spike level						average	Standard deviation	%RSD
	2 μg/kg			20 μg/kg					
α-ZAL	96.88	98.29	98.34	98.73	97.32	99.73	98.22	0.93	0.95
β-ZAL	105.08	103.12	104.06	102.35	105.13	104.52	104.04	1.02	0.98
α-ZOL	88.30	89.57	89.13	89.41	89.96	88.27	89.11	0.63	0.71
β-ZOL	82.68	80.31	85.18	82.18	83.75	81.15	82.54	1.61	1.95
ZAN	94.99	95.41	94.07	93.73	94.40	95.67	94.71	0.70	0.74
ZON	107.11	106.08	105.85	107.29	106.33	106.01	106.44	0.55	0.52

The linearity was evaluated by building external calibration curves for each compound using the ZEN-containing working solutions. The calibration curves were obtained by plotting the analyte peak area with respect to its seven differed concentrations. The respective concentration of the mixed standard solution was injected in triplicate, and then, the regression parameters were calculated. The results are shown in Table 2. The correlation coefficients (r^2^>0.999) were obtained for all compounds studied. These results demonstrate that an external standard calibration can be applied for quantitative purposes.

The sensitivity of the developed method was evaluated by determining the limits of detection (LOD) and quantification (LOQ) values. These values were calculated based on the response and slope of each regression equation at signal-to-noise ratios (S/N) of 3:1 and 10:1 respectively under the described chromatographic conditions. The LOD values were ranged across from 0.3 to 1.1 μg/kg for the different components, while the LOQ values ranged from 1.0 to 3.1 μg/kg. Detailed data are displayed in Table 2.

The method precision was then determined by measuring the intra- and inter-day precision. For the intra-day precision, six replicates of the mixed standard solutions were analyzed within a day, while for inter-day precision, the solutions were examined in triplicate for three consecutive days. The precision was expressed by the percentage of the relative standard deviation (%RSD). The overall %RSD values for the intra-days were < 3.8%, while the inter-day values were < 3.2% (see Table 3).

The accuracy was evaluated by adding the mixed standard solutions at two different concentrations (high: 20.0 μg/kg and low: 2.0 μg/kg) to the feed supplied from the Association of American Feed Control Official (AFFCO, 201625-Swine Feed, Medicated). The mixture was filtered and extracted using the developed IAC method. All tests were performed in triplicate. The results showed that the recovery rates were 82.5% -106.4% and also indicated that the accuracy level of the method was high. The details of the data are shown in Table 4.

Based on the above validation data, it was concluded that the proposed method provides excellent linearity, sensitivity, selectivity, accuracy, and precision for the simultaneous analysis of ZEN.

### Application of the developed method

ZEN was detected in all 75 animal feeds acquired from local farms that were analyzed in this study (Table 5). Each sample was analyzed in triplicate. Identification of the six compounds was by comparison of their retention times and mass spectrum with those of the standards and the pure compounds (Tables 1–4). ZEN was detected in 5 different types of animal feeds: dog, chicken, duck, pig and cattle, and ZEN was found in all of them. The reason for this lies in the fact that ZEN is expected to convert into and remain as other isomers due to oxidation and reduction in contamination feeds. Additionally, all ZEN was detected. One sample was detected from cattle, two samples were detected from chicken and duck, and three or four samples were detected from dog and pig. From the dog feed, β-ZAL and ZAN were each detected in the quantity of 2.40–18.73 μg/kg and 20.13–78.09 μg/kg. From chicken feed, α-ZAL was extracted in the quantity of 13.67–19.10 μg/kg and β-ZOL was detected lower than LOD. From duck feed, α-ZOL (4.19 μg/kg) and ZON (39.08–47.61 μg/kg) were extracted. For pig feed from which four samples were extracted, α-ZAL, β-ZAL, α-ZOL, ZAN and ZON were extracted. Detailed results are illustrated in Table 5.

**Table 5:** Determination of the contents (mg/kg) of 6 compounds in feed using the proposed method.

Feed type	No.*	No. of Detected	Concentration(μg/kg)					
			α-zearalanol (α-ZAL)	β-zearalanol (β-ZAL)	α-zearalenol (α-ZOL)	β-zearalenol (β-ZOL)	Zearalanone (ZAN)	Zearalenone (ZON)
Dog	16	3	-*	2.40~18.73	-	-	20.13~78.09	-
Chicken	13	2	13.67~19.10	-	-	N.D.*	-	-
Duck	15	2	-	-	4.19	-	-	39.08~47.61
Pig	17	4	2.31~2.48	3.11	N.D.	-	4.71~6.69	124.78
Cattle	14	1	-	2.45	-	-	-	N.D.

*No.: Number of total tested sample

-: Not Detected; N.D.: Lower than LOD

A simple qualitative and quantitative method for simultaneous determination of ZEN compounds from feeds was successfully developed and validated using LC-MS detection. The proposed method showed appropriate accuracy and precision, and it was successfully used for analyzing different types of feeds. The satisfactory results demonstrated that the LC-MS method provides a good alternative for routine analysis owing to its simplicity, specificity and sensitivity and to its potential to be applied as a reliable quality evaluation method for animal feed.

## References

[1] Qian M, Zhang H, Wu L, Jin N, Wang J, Jiang K. Simultaneous determination of zearalenone and its derivatives in edible vegetable oil by gel permeation chromatography and gas chromatography-triple quadrupole mass spectrometry. Food chemistry. 2015;166:23–8. doi: 10.1016/j.foodchem.2014.05.133 25053023 10.1016/j.foodchem.2014.05.133

[2] Ok HE, Choi SW, Kim M, Chun HS. HPLC and UPLC methods for the determination of zearalenone in noodles, cereal snacks and infant formula. Food chemistry. 2014;163:252–7. doi: 10.1016/j.foodchem.2014.04.111 24912723 10.1016/j.foodchem.2014.04.111

[3] Molina-Molina JM, Real M, Jimenez-Diaz I, Belhassen H, Hedhili A, Torne P, et al Assessment of estrogenic and anti-androgenic activities of the mycotoxin zearalenone and its metabolites using in vitro receptor-specific bioassays. Food and chemical toxicology: an international journal published for the British Industrial Biological Research Association. 2014;74:233–9. 25455890 10.1016/j.fct.2014.10.008

[4] Belhassen H, Jimenez-Diaz I, Ghali R, Ghorbel H, Molina-Molina JM, Olea N, et al Validation of a UHPLC-MS/MS method for quantification of zearalenone, alpha-zearalenol, beta-zearalenol, alpha-zearalanol, beta-zearalanol and zearalanone in human urine. Journal of chromatography B, Analytical technologies in the biomedical and life sciences. 2014;962:68–74. doi: 10.1016/j.jchromb.2014.05.019 24907545 10.1016/j.jchromb.2014.05.019

[5] Iqbal SZ, Nisar S, Asi MR, Jinap S. Natural incidence of aflatoxins, ochratoxin A and zearalenone in chicken meat and eggs. Food Control. 2014;43:98–103.

[6] Tanaka K, Sago Y, Zheng Y, Nakagawa H, Kushiro M. Mycotoxins in rice. International journal of food microbiology. 2007;119(1–2):59–66. doi: 10.1016/j.ijfoodmicro.2007.08.002 17913273 10.1016/j.ijfoodmicro.2007.08.002

[7] Geary PA, Chen G, Kimanya ME, Shirima CP, Oplatowska-Stachowiak M, Elliott CT, et al Determination of multi-mycotoxin occurrence in maize based porridges from selected regions of Tanzania by liquid chromatography tandem mass spectrometry (LC-MS/MS), a longitudinal study. Food Control. 2016;68:337–43.

[8] Cavaliere C, Foglia P, Guarino C, Motto M, Nazzari M, Samperi R, et al Mycotoxins produced by Fusarium genus in maize: determination by screening and confirmatory methods based on liquid chromatography tandem mass spectrometry. Food chemistry. 2007;105(2):700–10.

[9] De Baere S, Osselaere A, Devreese M, Vanhaecke L, De Backer P, Croubels S. Development of a liquid-chromatography tandem mass spectrometry and ultra-high-performance liquid chromatography high-resolution mass spectrometry method for the quantitative determination of zearalenone and its major metabolites in chicken and pig plasma. Analytica chimica acta. 2012;756:37–48. doi: 10.1016/j.aca.2012.10.027 23176738 10.1016/j.aca.2012.10.027

[10] Berthiller F, Sulyok M, Krska R, Schuhmacher R. Chromatographic methods for the simultaneous determination of mycotoxins and their conjugates in cereals. International journal of food microbiology. 2007;119(1–2):33–7. doi: 10.1016/j.ijfoodmicro.2007.07.022 17761332 10.1016/j.ijfoodmicro.2007.07.022

[11] Kinani S, Bouchonnet S, Bourcier S, Porcher JM, Ait-Aissa S. Study of the chemical derivatization of zearalenone and its metabolites for gas chromatography-mass spectrometry analysis of environmental samples. Journal of chromatography A. 2008;1190(1–2):307–15. doi: 10.1016/j.chroma.2008.02.115 18394636 10.1016/j.chroma.2008.02.115

[12] Tanaka T, Yoneda A, Inoue S, Sugiura Y, Ueno Y. Simultaneous determination of trichothecene mycotoxins and zearalenone in cereals by gas chromatography-mass spectrometry. Journal of chromatography A. 2000;882(1–2):23–8. 10895929 10.1016/s0021-9673(00)00063-7

[13] Ferreira I, Fernandes JO, Cunha SC. Optimization and validation of a method based in a QuEChERS procedure and gas chromatography–mass spectrometry for the determination of multi-mycotoxins in popcorn. Food Control. 2012;27(1):188–93.

[14] Onji Y, Aoki Y, Tani N, Umebayashi K, Kitada Y, Dohi Y. Direct analysis of several Fusarium mycotoxins in cereals by capillary gas chromatography–mass spectrometry. Journal of Chromatography A. 1998;815(1):59–65. 9718707 10.1016/s0021-9673(98)00357-4

[15] Branch SK. Guidelines from the International Conference on Harmonisation (ICH). Journal of Pharmaceutical and Biomedical Analysis. 2005;38(5):798–805. doi: 10.1016/j.jpba.2005.02.037 16076542 10.1016/j.jpba.2005.02.037

[16] Thompson M, Ellison S. and Wood R. Harmonized guidelines for single laboratory validation of methods of analysis. International Union of Pure and Applied Chemistry. 2002;74:835–55.

[17] Kim HJ, Jeong MH, Park HJ, Kim WC, Kim JE. Development of an immunoaffinity chromatography and HPLC-UV method for determination of 16 sulfonamides in feed. Food chemistry. 2016;196:1144–9. doi: 10.1016/j.foodchem.2015.10.014 26593600 10.1016/j.foodchem.2015.10.014

[18] Bae IK, Ham HM, Jeong MH, Kim DH, Kim HJ. Simultaneous determination of 15 phenolic compounds and caffeine in teas and mate using RP-HPLC/UV detection: method development and optimization of extraction process. Food chemistry. 2015;172:469–75. doi: 10.1016/j.foodchem.2014.09.050 25442580 10.1016/j.foodchem.2014.09.050

